# Inactivation of HIPK2 attenuates KRAS^G12D^ activity and prevents pancreatic tumorigenesis

**DOI:** 10.1186/s13046-024-03189-3

**Published:** 2024-09-28

**Authors:** Silvia Sozzi, Isabella Manni, Cristiana Ercolani, Maria Grazia Diodoro, Armando Bartolazzi, Francesco Spallotta, Giulia Piaggio, Laura Monteonofrio, Silvia Soddu, Cinzia Rinaldo, Davide Valente

**Affiliations:** 1 Unit of Cellular Networks and Molecular Therapeutic Targets, IRCCS Regina Elena National Cancer Institute, Rome, Italy; 2 Department of Science, Roma Tre University, Rome, Italy; 3 SAFU Unit, IRCCS Regina Elena National Cancer Institute, Rome, Italy; 4 Department of Pathology, IRCCS Regina Elena National Cancer Institute, Rome, Italy; 5 Pathology Research Laboratories, Sant’Andrea University Hospital, Rome, Italy; 6 Department of Biology and Biotechnologies “Charles Darwin”, Sapienza University, Rome, Italy; 7 Institute of Molecular Biology and Pathology (IBPM), National Research Council (CNR), c/o Sapienza University, Rome, Italy

**Keywords:** HIPK2, KRAS, Pancreatic tumorigenesis

## Abstract

**Background:**

Pancreatic ductal adenocarcinoma (PDAC) features KRAS mutations in approximately 90% of human cases and excessive stromal response, termed desmoplastic reaction. Oncogenic KRAS drives pancreatic carcinogenesis by acting on both epithelial cells and tumor microenvironment (TME). We have previously shown that Homeodomain-Interacting Protein Kinase 2 (HIPK2) cooperates with KRAS in sustaining ERK1/2 phosphorylation in human colorectal cancers. Here, we investigated whether HIPK2 contributes to oncogenic KRAS-driven tumorigenesis in vivo, in the onset of pancreatic cancer.

**Methods:**

We employed an extensively characterized model of KRAS^G12D^-dependent preinvasive PDAC, the *Pdx1-Cre;LSL-KRas*^*G12D/*+^ (KC) mice. In these mice, HIPK2 was inhibited by genetic knockout in the pancreatic epithelial cells (KCH^−/−^) or by pharmacologic inactivation with the small molecule 5-IodoTubercidin (5-ITu). The development of preneoplastic acinar-to-ductal metaplasia (ADM), intraepithelial neoplasia (PanIN), and their associated desmoplastic reaction were analyzed.

**Results:**

In *Hipk2*-KO mice (KCH^−/−^), ERK phosphorylation was lowered, the appearance of ADM was slowed down, and both the number and pathologic grade of PanIN were reduced compared to *Hipk2*-WT KC mice. The pancreatic lesion phenotype in KCH^−/−^ mice was characterized by abundant collagen fibers and reduced number of αSMA^+^ and pSTAT3^+^ desmoplastic cells. These features were reminiscent of the recently described human “deserted” sub-TME, poor in cells, rich in matrix, and associated with tumor differentiation. In contrast, the desmoplastic reaction of KC mice resembled the “reactive” sub-TME, rich in stromal cells and associated with tumor progression. These observations were confirmed by the pharmacologic inhibition of HIPK2 in KC mice.

**Conclusion:**

This study demonstrates that HIPK2 inhibition weakens oncogenic KRAS activity and pancreatic tumorigenesis providing a rationale for testing HIPK2 inhibitors to mitigate the incidence of PDAC development in high-risk individuals.

**Graphical Abstract:**

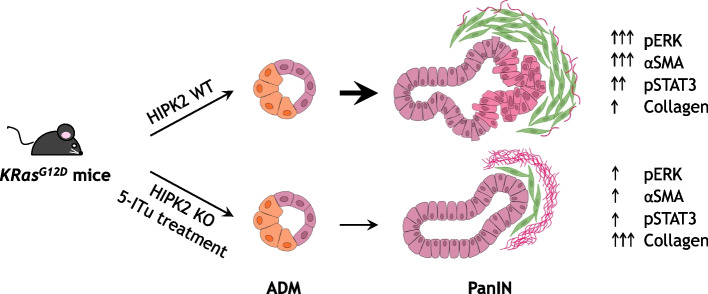

**Supplementary Information:**

The online version contains supplementary material available at 10.1186/s13046-024-03189-3.

## Background

Pancreatic ductal adenocarcinoma (PDAC) is among the most refractory malignancies and carries a poor prognosis. Despite advances in targeted- and immuno-therapies, PDAC has remained one of the few human cancers that has not shown a reduction in mortality rates over time [1]. PDACs arise from stepwise progression of pancreatic intraepithelial neoplasms (PanINs) through a slow process that can take many years before turning into invasive cancer [2]. In the last two decades, different high-risk conditions of PDAC predisposition have been recognized [3–5] prospecting opportunities for prevention. Thus, in addition to the development of new therapeutic approaches, novel targets and strategies to prevent or delay the occurrence of pancreatic cancers before they evolve to incurable stages are under investigation [6].

The Kirsten RAS (*KRAS*) gene mutations are oncogenic drivers often present in early PanINs and their frequency exceeds 90% in human PDAC [7]. In several genetically engineered mouse models, oncogenic KRAS expression in pancreatic epithelial cells has been shown to be necessary for disease initiation and maintenance through cell-autonomous actions (*e.g*., regulation of cell proliferation, differentiation, metabolism, and replicative senescence) and non-cell autonomous remodeling of both premalignant and tumor microenvironment (TME) (reviewed in [8]). In response to tissue injury, such as acute or chronic inflammation, acinar cells downregulate the expression of digestive enzymes, becoming protected from them, and differentiate into duct-like cells through a process called acinar-to-ductal metaplasia (ADM). ADM is a reversible process and the acinar parenchyma is re-established upon damage resolution. However, in the presence of oncogenic KRAS and activation of its downstream pathways, ADM becomes irreversible and the duct-like cells undergo neoplastic transformation into PanINs [9–12]. Together with these cell-autonomous activities, oncogenic KRAS induces the production of cytokines, chemokines, and growth factors that shape the robust desmoplastic response typical of pancreatic tumorigenesis (reviewed in [8]). In the early PanIN precursor microenvironment, oncogenic KRAS has been shown to trigger the activation/reprogramming of fibroblasts, which express α-smooth muscle actin (αSMA) depending on the signal transducer and activator of transcription 3 (STAT3), and the subsequent stimulation of tumor-promoting inflammatory program [13].

Homeodomain interacting protein kinase 2 (HIPK2) is an evolutionarily conserved tyrosine-regulated serine/threonine kinase that contributes to various physiological and pathological conditions, including regulation of morphogenesis, DNA damage response, cell death, cell proliferation, and tissue fibrosis [14, 15]. Upon activation, HIPK2 modulates, in a context-dependent manner, gene transcription and numerous signal transduction pathways, including those primarily involved in tumorigenesis, such as TP53, TGF-β/SMAD, WNT/β-catenin, and JNK/STAT [16–18]. Recently, HIPK2 has been shown to cooperate with KRAS signaling and associate with human colorectal cancer progression. In mutant KRAS-carrying colon cancer cells, HIPK2 depletion impairs phosphorylation of extracellular signal-regulated kinases 1 and 2 (ERK1/2) and tumor growth in a xenograft model [19]. At molecular level, HIPK2 does not reduce RAS activity (*i.e*., GTP-bound RAS) but works at the level of the mitogen activated protein kinase (MAPK) cascade. In particular, HIPK2 physically participates in the downstream RAS complex and contributes to the phosphorylation of RAF1-Ser338, BRAF-Ser446, MEK, and ERK1/2 [19]. HIPK2 requirement for the maintenance of ERK phosphorylation was also demonstrated in mouse cardiomyocytes and in basal cardiac function [20]. These data suggest that HIPK2 is a novel player in the RAS signaling network and its presence contributes to phosphorylation/activation of, at least, the MAPK pathway. However, it has not been tested whether HIPK2 cooperates with KRAS-driven tumorigenicity in vivo, employing tissue-specific expression of oncogenic KRAS and HIPK2 knockout (KO).

In the current study, we investigated how HIPK2 contributes to oncogenic KRAS-driven tumorigenesis in the onset of pancreatic cancer. We chose pancreatic tumorigenesis for two main reasons: one is the early and very high frequency of KRAS mutations in human PDAC, the second is the extensively characterized mouse model that express mutant KRAS^G12D^ in epithelial pancreatic cells (*i.e.,* KC mice) and that recapitulate most of the features of pancreatic tumorigenesis, from preneoplastic lesions to invasive cancer [21]. Here, we provide evidence that both genetic and pharmacological inactivation of HIPK2 in KC mice weakens oncogenic KRAS, desmoplastic reaction, and pancreatic tumorigenesis.

## Materials and methods

### Cell culture

Human PDAC cell lines (*i.e*., PANC1, PaTu 8988T, ASPC-1, C5M2, HPAF II, KP4) and the near-normal human pancreatic ductal cell line (HPDE) were kindly provided by Dr. Paola Nisticò (Unit of Tumor Immunology and Immunotherapy, Regina Elena National Cancer Institute, Rome Italy). Cells were maintained in growth medium containing 10% fetal bovine serum (Gibco), Glutamax and Penicillin/Streptomycin (Gibco) at 37° C in humidified incubator with 5% CO_2_. HPDE (RRID:CVCL_4376), PANC1 (RRID:CVCL_0480) and HPAF II (RRID:CVCL_0313) were maintained in RPMI 1640 Medium, PaTu 8988T (RRID:CVCL_1847) in DMEM-High Glucose, AsPC1 (RRID:CVCL_0152) and C5M2 in DMEM-Low Glucose, and KP4 (RRID:CVCL_1338) in IMDM (all from Gibco).

### Western Blotting (WB)

Whole protein lysates were obtained from frozen pancreatic tissue using GentleMacs dissociator (Miltenyi Biotec) or frozen cell pellet using lysis buffer [50 mmol/L Tris–HCl pH 8, 150 mmol/L NaCl, 0.5% sodium deoxycholate, 0.1% SDS, 1% IGEPAL, and 1 mmol/L EDTA] supplemented with protease-inhibitor mix (Roche Complete) and Halt Phosphatase Inhibitor Cocktail (Thermo Fisher Scientific), quantified by Bio-Rad Protein assay Dye (Bio-Rad Laboratories, Inc.), separated by SDS-PAGE onto 4 to 12% gels (Bolt, Invitrogen) and then transferred onto nitrocellulose membranes (Bio-Rad Laboratories, Inc.). After blocking with 5% skimmed dry milk (Bio-Rad Laboratories, Inc.), membranes were incubated with primary and secondary Abs enlisted in Supplementary Table S1. Immunoreactions were detected with ECL WB Detection System (GE Healthcare).

### Human samples

Pancreatic formalin-fixed paraffin-embedded (FFPE) tissues from 44 patients with pancreatic tumors were from the IRE Biobank. The clinicopathologic characteristics of PDAC patient cohort, including age at surgery, sex, site, tumor size, nodal status, grade, metastasis, and HIPK2 positive cells are reported in Table 1. Tumors were staged according to the American Joint Committee on Cancer Staging Manual. The Institutional Ethics Committee (Comitato Etico Centrale I.R.C.C.S. Lazio, Sezione IRCCS I.F.O.—Fondazione G.B. Bietti) approved this study (CE/694/15) and all patients signed their informed consent for participation.

**Table 1:** Clinico-pathologic characteristics of PDAC patient cohort

Characteristics	N	%
**Number of patients**	44	
**Age at surgery** (years) Mean ± SD	65.8 ± 9.5	
**Sex** (Male)	28	63.6
**Site**		
Head	9	20.5
Body	30	68.2
Papilla	5	11.4
**Tumor size**		
T1	1	2.3
T2	8	18.2
T3	32	72.7
T4	1	2.3
N.D	2	4.5
**Nodal status**		
N-	24	54.5
N +	20	45.5
N.D	2	4.5
**Grade**		
1	3	6.8
2	18	40.9
3	23	52.3
**Metastasis**		
Absent	43	97.7
Present	1	2.3
**HIPK2**^**+**^** cells**		
0	5	11.4
≤ 5	5	11.4
5 ≤ 20	8	18.2
20 ≤ 40	16	36.4
≥ 40	10	22.7

*Abbreviations*: *SD* standard deviation, *N*- node negative, *N* + node positive, *N.D.* non-determined

### Animals and treatments

Animals were housed at the IRE animal facility (SAFU). All animal studies were approved by the Institutional Animal Care of IRE and by the Government Committee of National Minister of Health (ethics review numbers: 1056/2015-PR and 362/2021) and conducted according to EU Directive 2010/63/EU and Italian D.L. 2614/2014 for animal experiments following the Institutional Guidelines for Animal Care and Welfare. The following mouse strains were used: B6.Cg-Tg (ACTFLPe)9205Dym/J (RRID:IMSR_JAX:005703) purchased from The Jackson Laboratories; C57BL/6NTac-Hipk2tm2a(EUCOMM)Hmgu/Cnrm (RRID:IMSR_EM:05113) purchased from the EUCOMM Monterotondo; C57BL/6 Pdx1-Cre mice and LoxSTOPLox (LSL)-KRas^G12D/+^ [21] kindly provided by Prof. Francesco Novelli (University of Turin); FVB Pdx1-Cre;LSL-KRas^G12D/+^ [22] from I.M. and G.P.. Inclusion criteria: homozygous male and female Pdx1-Cre;LSL-KRas^G12D/+^;Hipk2^WT/WT^ (KC) and Pdx1-Cre;LSL-KRas^G12D/+^;Hipk2^flox/flox^ (KCH^−/−^). Exclusion criteria: heterozygous Pdx1-Cre;LSL-KRas^G12D/+^;Hipk2^WT/flox^. Age and sex are reported in Table 2. For treatment with 5-Iodotubercidin (5-ITu) (Sigma-Aldrich), nine-weeks old FVB Pdx1-Cre;LSL-KRas^G12D/+^ mice (six males and six females) were randomly subdivided into two groups (three males and three females per group). One group was injected intraperitoneally (i.p.) twice a week for seven weeks with 0.25mg/Kg BW of 5-ITu, as described [23]; littermate control group was injected with an equivalent amount of the 5-ITu solvent, DMSO (5% vol:vol).

**Table 2:** Clinical spectrum of disease in KC and KCH^−/−^ mice

**Age** (weeks)	**Sex**	**Genotype**	**Pancreas histology**	**Muc.papil**	**Lymph. prol.dis**	**Other pathological features**
20	♂	KC	ADM; PanIN-1 and 2	+	-	splenomegaly
26	♀	KC	ADM; PanIN-1, 2, and rare 3	+	+	splenomegaly
28	♂	KC	ADM; PanIN-1 and 2, rare 3	+	-	-
28	♀	KC	ADM; PanIN-1	+	-	-
28	♂	KC	PanIN-3; early PDAC	-	-	-
29	♀	KC	ADM; PanIN-1 and 2	+	-	-
30	♂	KC	ADM; PAnIN-1, rare 2	+	-	-
30	♂	KC	ADM; PanIN-1, rare 3	-	+	lung mass
31	♀	KC	ADM; PanIN-1, 2 and 3	+	-	splenomegaly
44	♂	KC	PanIN-3; cystic PDAC	+	-	-
44	♀	KC	ADM; PanIN-1 and 2	+	-	splenomegaly
45	♂	KC	ADM; PanIN-3	+	+	lung mass
13	♀	KCH^−/−^	normal pancreatic tissue	-	+	thymic and lung masses
14	♀	KCH^−/−^	normal pancreatic tissue	-	+	thymic mass
14	♂	KCH^−/−^	ADM; PanIN-1	-	+	splenomegaly; lung mass
18	♂	KCH^−/−^	normal pancreatic tissue	+	-	splenomegaly
22	♀	KCH^−/−^	normal pancreatic tissue	+	-	-
22	♂	KCH^−/−^	ADM; PanIN-1	+	-	-
28	♀	KCH^−/−^	ADM; PanIN-1 and 2	+	-	-
30	♂	KCH^−/−^	ADM; PanIN-1, 2, and 3	+	-	-
36	♂	KCH^−/−^	ADM; PanIN-1 and 2	+	-	-
43	♂	KCH^−/−^	ADM; PanIN-1	+	-	-
43	♂	KCH^−/−^	ADM; PanIN-1, rare 2 and 3	+	-	bladder mass
43	♂	KCH^−/−^	ADM; PanIN-1	+	-	-
45	♀	KCH^−/−^	ADM; PanIN-3	+	+	lung mass
51	♂	KCH^−/−^	ADM; PanIN-1	+	-	-

*Muc. papil.* mucocutaneous papilloma, *Lymph. prol. dis* lymphoproliferative disease

### Organoid cultures and treatments

Murine pancreatic organoids were obtained from KC and KCH^−/−^ mice following the protocol described in Broutier et al. [24]. Organoids were plated on coverslip and cultured in PancreaCult™ Organoid Growth Medium (STEMCELL Technologies).

To test KRAS pathway activation, KC and KCH^−/−^ organoids were cultured for 5 h in AdDMEM supplemented with 1 μM 5-ITu or an equivalent amount of DMSO, as a control. After the pre-treatment, organoids were treated or not with 50 ng/ml mouse Epidermal Growth Factor (mEGF) for 30 min in the presence of 5-ITu or DMSO. Following treatment, organoids were fixed in 3.7% formaldehyde for 10 min at room temperature, then permeabilized with 0.25% Triton X-100 for 15 min. Organoids were blocked in 5% Bovine Serum Albumin for 1 h and incubated overnight at 4°C with the anti-phospho-ERK (pERK) Ab (Cell Signaling Technology, Supplementary Table S1). After washing, organoids were incubated with the Alexa Fluor™ 488-conjugated anti-rabbit secondary Ab (Supplementary Table S1) for 1 h at 37°C. Nuclei were counterstained with 1 µg/ml Hoechst 33342 (Sigma-Aldrich) for 10 min. Coverslips were then mounted on slides using VECTASHIELD® Antifade Mounting Medium (Vector Laboratories).

### Genotyping

Mice and organoids were genotyped by PCR. DNA was obtained by incubating tissues in lysing buffer [100mmol/L Tris–HCl Ph8, 0.5% IGEPAL, 0.5% Tween 20] plus Proteinase K (Invitrogen) overnight at 55° C, then 1h at 85° C to inactivate Proteinase K. PCR was performed with an Applied Biosystem 9700 thermocycler using Promega G2 Taq following manufacturer’s instructions with primers reported in Supplementary Table S1.

### Real Time-PCR (RT-qPCR)

RNA extraction was performed on frozen tissues in Trizol (Invitrogen) using GentleMACS Dissociator (Miltenyi Biotech), then RNA was isolated according to the Trizol manufacturer’s instructions. Reverse transcription reactions were conducted using a M-MLV Reverse Transcriptase (Invitrogen). Samples for RT-qPCR were prepared with 1 × SYBR Green PCR Master Mix (Applied Biosystems) and different primers (enlisted in Supplementary Table S1). All primers were optimized for amplification under reaction conditions as follow: 95° C for 10 min followed by 40 cycles of 95° C for 15 s and 60° C for 1 min. Dissociation curve analyses were performed for all samples after completion of the amplification protocol. *Gapdh* was used as housekeeping gene expression control.

### Histopathological analyses

Pancreatic tissue from euthanized animals was fixed in formalin and processed to obtain conventional FFPE tissue blocks. H&E, Masson’s trichrome, and immunohistochemistry (IHC) were performed according to the standard histological procedures. Picrosirius red (PR) staining (BiO-Optica) was performed, as indicated, in combination or not with Mayer's Hemalum counterstaining according to manufacturer’s instructions. IHC on FFPE-derived murine tissue sections was performed with the primary Abs indicated in Supplementary Table S1. Briefly, FFPE tissue blocks were sectioned (3µm-thick) and then submitted to deparaffinization and rehydration. Antigen-retrieval microwave treatment (0.01 M citrate buffer pH 6.0) was applied for 5 min, at 750 W, and for 3 min at 180 W. Immune-reactions were visualized by using EnVision™ FLEX kit (EnVision™ FLEX; Agilent). For morphological evaluation of ADM, a minimum of 5 randomly chosen fields (at 10 × magnification) of H&E were counted for each pancreas. For PanIN evaluation, a minimum of 50 normal/pathological total ducts were counted for each pancreas. Each duct was classified as normal, PanIN-1, -2, or -3 based on the classification consensus [25]. Randomly selected, non-overlapping images (5 × objective) were taken for each slide using LAS X v3.7.5.24914 software on Leica DMIL-led microscope with a FlexaCam C1 v1.10c camera. The scores of IHC signals for the reported Abs were assessed both on digital images and directly at the optical microscope and assigned blinded to mice genotype by three investigators (A.B., S.S., and D.V.). At least 15 randomly selected fields were taken for each pancreas for the analyses. The proliferation index was assessed counting Ki67 ductal positive cells of ADM or PanIN ducts in KC and KCH^−/−^ samples. A total of at least 800 cells were counted for each condition. For sub-TME categorization, each pancreatic lesion was classified as deserted or reactive based on both αSMA and PR signals [26].

Histopathological analyses of human samples were performed on H&E-stained slides. For IHC, the anti-HIPK2 (5C6) rat monoclonal Ab [27], kindly provided by Prof. M. Lienhard Schmitz (Giessen University, Germany) was diluted 1:50, incubated at room temperature for 30 min, and detected by an anti-polyvalent diaminobenzidine staining system containing both blocking reagent and secondary Ab (ULTRATEK HRP; ScyTek Laboratories, Inc.), according to the manufacturer's protocol, as previously described [19]. The percentages of nuclear HIPK2 positive (HIPK2^+^) ductal cells (both normal and cancer ducts) were evaluated independently by two blinded investigators (M.G.D. and C.E.) by manually counting more than 200 cells per sample at high magnification (40x).

### Statistical analysis

Statistical analyses were performed using GraphPad Prism v.9 (GraphPad). Normal distribution of data was assessed using Shapiro Wilk’s and Kolmogorov–Smirnov tests in Prism; differences between groups were examined using 2-tailed Student’s t-test, ANOVA test, Fisher’s exact test, when appropriate, as indicated in the relative Figure’s Legends. Statistical significance was set at *P* < 0.05. Sample size (n) and replication are indicated in the relative figure's legends. The data generated in this study are available on request from the corresponding author.

## Results

### *HIPK2* is expressed in human PDAC

**Fig. 1:**
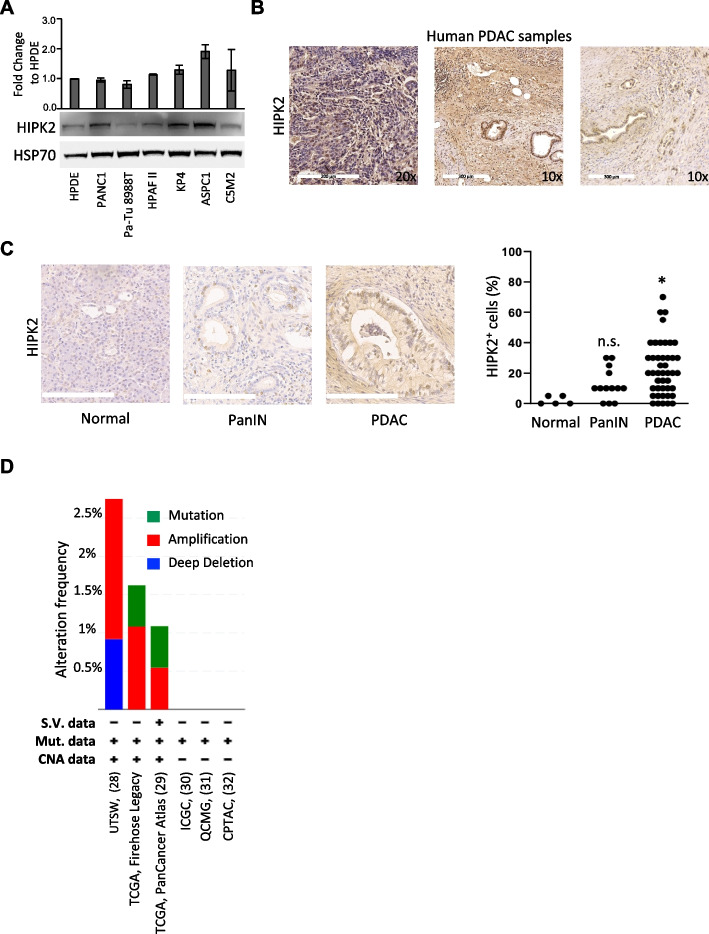
HIPK2 is expressed in human PDACs. **A** WB analysis of HIPK2 in whole cell lysates of the indicated human pancreatic and PDAC-derived cell lines. HSP70 was used as loading control. The histogram indicates the mean ± standard error of HIPK2 fold change expression relative to HPDE in three independent experiments. **B** Representative images of IHC for HIPK2 in three different human PDAC samples. **C** Representative images for HIPK2 immunostaining in normal, PanIN and PDAC ducts are reported on the left. Scale bars are 200 µm. Quantification of the percentage of HIPK2^+^ cells in the indicated tissues is reported as dot plot on the right. ANOVA test with Tukey’s multiple comparison test, * *P* < 0.05, n.s.: not significant. **D** TCGA analysis of the *HIPK2* gene alteration frequency as reported in cBioPortal (https://www.cbioportal.org/). S.V.: Structural variant; Mut.: Mutation. CNA: Copy Number Alteration. Each histogram shows the frequency of HIPK2 gene alteration in the indicated dataset

To investigate the contribution of HIPK2 to oncogenic KRAS-driven tumorigenesis in the onset of pancreatic cancer, we first assessed the expression of HIPK2 in human PDACs by WB, employing highly specific anti-HIPK2 Ab previously validated on human *HIPK2*-null cells [27] and tissue microarrays [19]. We performed WB analyses on whole cell lysates from PDAC-derived cell lines (*i.e*., PANC1, PaTu8988T, HPAF II, KP4, C5M2, and ASPC1) and near-normal human pancreatic duct epithelial cells (HPDE) and found that HIPK2 is expressed in cells of all lines at similar level (Fig. 1A). Next, we analyzed HIPK2 expression by IHC in 44 surgically resected human PDAC samples from IRE Biobank (Table 1). Although the small number of PDAC samples did not allow to establish any type of association with specific clinicopathological features, the expression of HIPK2 was detected in the majority of PDACs (88.6%), with the expected staining and an overall intensity in the positive cells similar in most samples (Fig. 1B). Quantification of the percentage of HIPK2^+^ ductal cells present in our samples ranged from 0 (11.4% of cases) to more than 40% (22.7% of cases), with the highest frequency of cases (36.4%) showing a percentage of HIPK2^+^ cells ranging from 20 to 40% (Fig. 1C and Table 1). When present in the IHC samples, the PanIN lesions showed a comparable pattern of HIPK2 staining with a percentage of HIPK2^+^ cells ranging from 0 to 30% (Fig. 1C). These results are consistent with data retrieved from cancer genome databases (cBioPortal, COSMIC), showing that the *HIPK2* gene is maintained in the WT form in human pancreatic cancers. Indeed, *HIPK2* is present with low alteration frequency (no more than 3%), among which amplification is found as the most abundant variation (Fig. 1D) [28–32]. Thus, similarly to what was previously observed in human colorectal cancers, these data show that HIPK2 expression is maintained during pancreatic tumorigenesis.

### Genetic ablation of *Hipk2* in KRas^G12D^-expressing murine pancreas reduces ERK phosphorylation

**Fig. 2:**
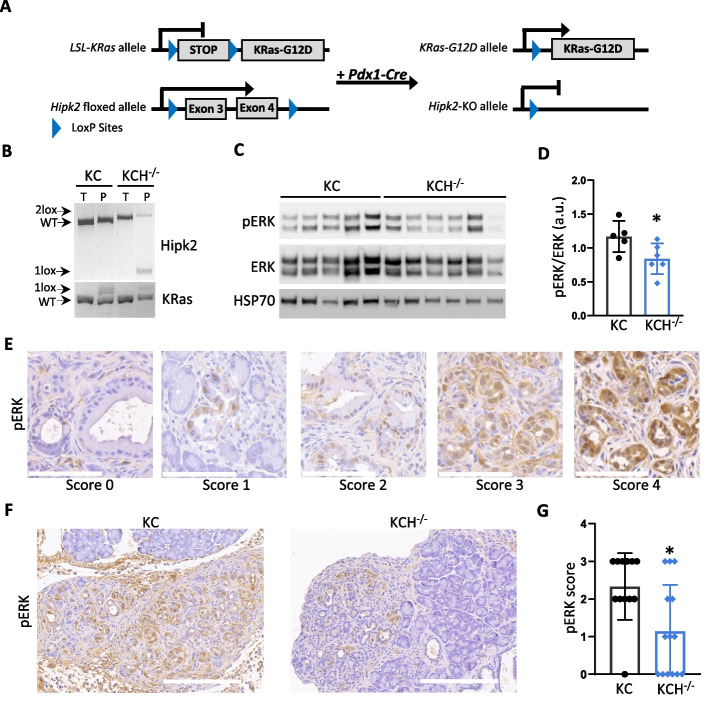
The levels of pERK are reduced in KCH^−/−^ pancreata. **A** Schematic representation of LSL-KRas^G12D/+^ and *Hipk2*^*flox/flox*^ recombinant alleles. **B** Representative genotyping analysis for *Hipk2*^*flox*^ and *LSL-KRas*^*G12D*^ alleles of tail (T) and pancreas (P) from *Pdx1-Cre;LSL-KRas*^G12D/+^ (KC) and *Pdx1-Cre;LSL-KRas*^*G12D/*+^*;Hipk2*^*flox/flox*^ (KCH^−/−^) mice. Floxed (2lox) and recombined (1lox) alleles are indicated by arrows. **C** WB analysis of pERK levels in KC and KCH^−/−^ pancreata. HSP70 was used as loading control. **D** Normalized levels of pERK represented as ratio of pERK and total ERK densitometry values. Mean ± Standard Deviation (SD) is reported. Student’s t-test, * *P* < 0.05. **E** Representative IHC images for each pERK score. Scale bars are 100 µm. **F** Representative IHC images of pERK in KC and KCH^−/−^ pancreata. Scale bars are 200 µm. **G** Scatter plot bar of pERK score in all KC and KCH^−/−^ mice. Mean ± SD is reported at the bottom. Mann–Whitney’s test, * *P* < 0.05

To interrogate the functional contribution of HIPK2 in oncogenic KRAS-driven pancreatic carcinogenesis, we first evaluated the phenotype of pancreas-specific KO of the *Hipk2* gene by analyzing *Pdx1-Cre;Hipk2*^*flox/flox*^ mice generated by crossing *Hipk2*^*flox/flox*^ mice with *Pdx1-Cre* mice, that express CRE recombinase under the control of the pancreatic specific Pdx1 promoter [21]. *Pdx1-Cre;Hipk2*^*flox/flox*^ mice were born at the expected frequency and successful recombination of the *Hipk2* gene in the pancreas was assessed by PCR (Supplementary Fig. S1A). We observed the *Pdx1-Cre;Hipk2*^*flox/flox*^ mice for up to 72 weeks of age detecting no signs of distress throughout their life. Histological analysis of pancreata by H&E staining showed no significant difference between *Pdx1-Cre;Hipk2*^*flox/flox*^ and *Pdx1-Cre;Hipk2*^*WT/WT*^ mice (Supplementary Fig. S1B), suggesting that *Hipk2*-KO does not impair pancreatic development. Next, we crossed our *Pdx1-Cre;Hipk2*^*flox/flox*^ mice with a well-established model of KRas-driven preinvasive and invasive ductal pancreatic cancer, the *Pdx1-Cre;LSL-KRas*^*G12D/*+^ (KC) mice [21] (Fig. 2A). In the resulting *Pdx1-Cre;LSL-KRas*^*G12D/*+^*;Hipk2*^*flox/flox*^ mice (from here on, KCH^−/−^), expression of CRE recombinase under control of the Pdx1 promoter induces the ablation of HIPK2 in the same pancreatic epithelial cells that express KRAS^G12D^ (Fig. 2B).

Next, to evaluate whether HIPK2 cooperates with oncogenic KRAS activity in KC mice, we analyzed the expression levels of phosphorylated ERK1/2 (pERK) by WB on whole lysates of pancreata obtained from five KC and six KCH^−/−^ mice randomly selected. A mild, but statistically significant reduction of pERK was observed in KCH^−/−^ mice compared to KC (Fig. 2 C, D). This result was confirmed by the IHCs we performed on each pancreas of both mouse lines. As shown in Figs. 2E-G, IHC revealed a significantly weaker expression of pERK in KCH^−/−^ mice than in KC, indicating that *Hipk2*-KO impairs oncogenic KRAS signaling in murine pancreas.

### *Hipk2*-KO attenuates pancreatic tumorigenesis in KC mice

**Fig. 3:**
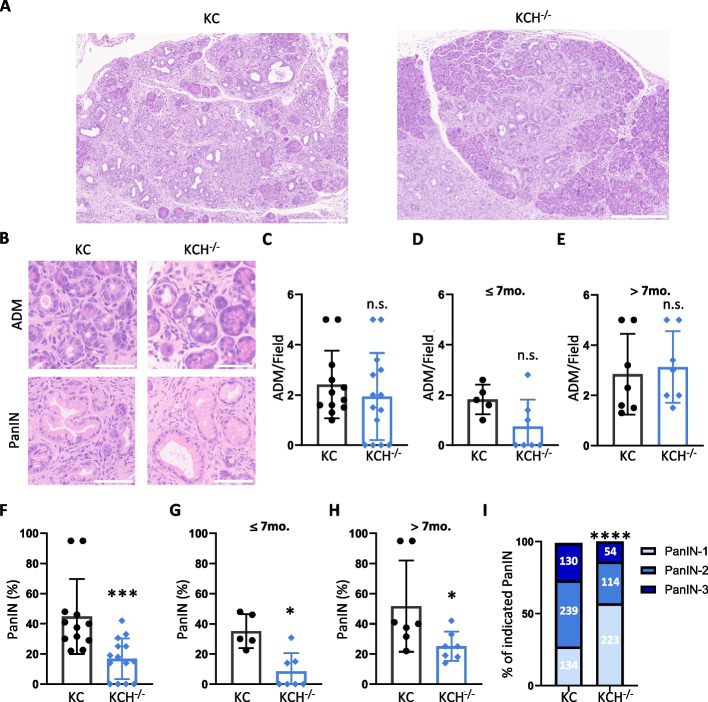
Depletion of HIPK2 reduces pancreatic tumorigenesis in KC mice. **A** Representative images of pancreas slices from KC and KCH^−/−^ stained with H&E. PanIN and ADM lesions are visible. Scale bars are 300 µm. **B** Representative images of ADM and PanIN in KC and KCH^−/−^ pancreata. Bar is 50 µm. **C** ADM in each visual field were counted in 12 KC and 14 KCH^−/−^ pancreatic samples stained by H&E. Corresponding quantification for each mouse is reported in the scatter plot bar. Mean ± SD, Mann–Whitney’s test, n.s. not significant. **D**-**E** Data in B were divided into two subgroups based on the mouse age at the euthanization to obtain ADM histograms in mice younger or older than 7 months ($\le$ 7mo and $>$ 7mo), respectively. Mean ± SD, Mann–Whitney’s test, n.s. not significant. **F** The percentage of PanINs relative to unaffected ducts was evaluated on the same samples described in B and shown as scatter plot bar. Mean ± SD, Mann–Whitney’s test, *** *P* < 0.001. **G**-**H** Data in E were divided in two subgroups ($\le$ 7mo and $>$ 7mo) as described above. Mean ± SD, Mann–Whitney’s test, * *P* < 0.05. **I**, PanINs present in the H&E samples were counted based on their grade. The percentage of each PanIN grade is shown and the number of counted PanINs is indicated inside the histograms. Fisher’s exact test, **** *P* < 0.0001

We next evaluated the contribution of *Hipk2*-KO in the development of ADM, PanIN, and PDAC. In the C57BL/6 strain, KC mice develop spontaneous PanIN within 9 to 18 weeks, while their progression to invasive PDAC is rare (3.4%) [21]. In addition, KC mice are reported to develop undesired phenotypes such as mucocutaneous papilloma and lymphoproliferative disease [21, 33, 34]. In our C57BL/6 strain, both KC and KCH^−/−^ mice developed mucocutaneous papilloma with similar high incidence (> 90%) and lymphoproliferative disease in a few animals (Table 2). Papilloma occurred on muzzle and perineum in both male and female mice (Supplementary Fig. S2A). Although benign in nature, as shown by histological analysis (Supplementary Fig. S2B), these undesired phenotypes necessitated the euthanization of affected animals, with endpoints ranging from 13 to 51 weeks (Table 2), biasing the survival curves related to eventual, late PDAC development. Indeed, among 12 KC and 14 KCH^−/−^ mice, only one KC showed abdominal swelling, a symptom of PDAC, and was euthanized at 28 weeks of age for this reason (Table 2). Thus, we focus our study on microscopic evaluation of pancreata following the Consensus Report and Recommendations for genetically engineered mouse models of pancreatic exocrine cancer [25]. We first evaluated the spontaneous appearance and the amount of ADM by H&E staining (Fig. 3A, B). Pooling data of all pancreata from KC mice and those from KCH^−/−^ mice, independently of the age of euthanization, there was no significant difference between *Hipk2*-WT and -KO mice in the number of ADM lesions (Fig. 3B, upper panels and 3C). However, when we subdivided the mice into two groups, *i.e*., those euthanized between 13 and 28 weeks of age ($\le$ 7mo) and those between 29 and 51 ($>$ 7mo), defining 7mo as the age at which KC mice develop preneoplastic lesions in more than 60% of the sample [21, 22], even if not statistically significant, a trend of delay in ADM appearance was detected in KCH^−/−^ mice compared with KC (Fig. 3D). This delay was recovered in the $>$ 7mo group (Fig. 3E), suggesting that *Hipk2*-KO in epithelial cells slows down ADM formation, but does not prevent it. Next, on the same H&E tissue sections, we assessed the number and the grade of PanINs. PanINs developed in both mouse lines (Fig. 3B, lower panels), but their number related to total ducts was significantly smaller in *Hipk2*-KO mice than in *Hipk2*-WT mice (Fig. 3F). This difference was present both on pooled data and upon subdivision into the two age-related groups ($\le$ 7mo and $>$ 7mo) (Fig. 3F-H). The pathological grade of PanINs was significantly lower in KCH^−/−^ mice than in KC (Fig. 3I). In addition, proliferation index, apoptosis, and lymphocyte infiltration were evaluated. In the ADM, the proliferation index (*i.e.,* Ki67^+^ cells) was similar in KC and KCH^−/−^ mice, while in the PanIN, Ki67^+^ ductal cells were significantly lower in KCH^−/−^ (Supplementary Fig. S3). In contrast, very low amount with no significant differences among the samples were detected both in apoptosis appearance (*i.e.,* Cleaved Caspase 3 positive cells) and lymphocyte infiltration (*i.e.,* CD3 positive cells) (data not shown). These findings indicate that *Hipk2*-KO delays ADM formation and reduces their progression into PanINs.

### *Hipk2*-KO modifies the pancreatic microenvironment shaped by oncogenic KRAS

**Fig. 4:**
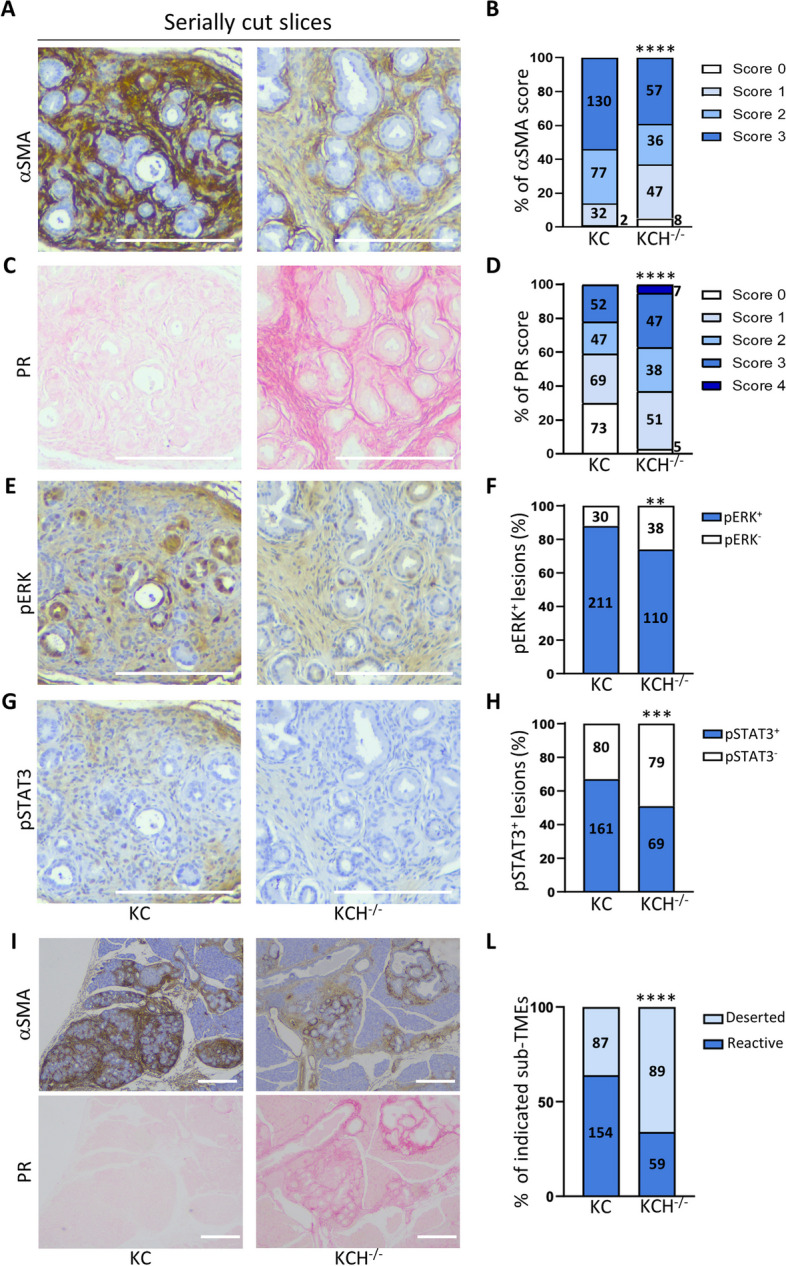
Loss of HIPK2 determines different oncogenic KRAS sub-TMEs. Pancreatic lesions of all KC and KCH^−/−^ mice were analyzed with the indicated staining by serially cut slices. Scale bars are 200 µm **A**, **B**, Representative images of IHC for aSMA are shown in A; the percentages of the different score staining intensity (from 0 to 3) are reported in B and the number of lesions counted for each score is indicated inside the histograms; Fisher’s exact test, **** *P* < 0.0001. **C**, **D** Representative images of PR staining are shown in C; the percentages of the different score staining intensity (from 0 to 4) are reported in D and the number of lesions counted for each score is indicated inside the histograms; Fisher’s exact test, **** *P* < 0.0001. **E**, **F** Representative images of IHC for pERK are shown in E; the percentages of lesions positive for pERK (pERK^+^) are reported as histograms in F, Fisher’s exact test, ** *P* < 0.01. **G**, **H** Representative images of IHC for pSTAT3 are shown in G; the percentages of lesions positive for pSTAT3 (pSTAT3.^+^) are reported as histograms in H, Fisher’s exact test, *** *P* < 0.001. **I** Representative images of αSMA (upper images) and Picrosirius Red (lower images) showing reactive and deserted sub-TMEs in the indicated mice. **L** The percentages of lesions with reactive and deserted sub-TME are reported and the number of lesions counted for each sub-TME is indicated inside the histograms. Fisher’s exact test, **** *P* < 0.0001

To further examine the features of PanINs that developed in KC and KCH^−/−^ mice, we analyzed the desmoplastic reaction that coevolves with KRAS-driven transformation of the pancreatic epithelial cells [20]. We histologically evaluated four samples of serially cut slices from each mouse carrying pancreatic lesions (*i.e*., 12 out of 12 KC mice and 10 out of 14 KCH^−/−^ mice). For each mouse, the slices were stained for αSMA as a marker of fibroblast activation, Picrosirius red (PR) for collagen fibers staining, pERK as readout of KRAS activity, and phosphorylated STAT3 (pSTAT3) as a marker of fibroinflammatory response [13]. We observed a statistically significant higher intensity for αSMA (Fig. 4A, B) and lower for PR staining (Fig. 4C, D) in the lesions of KC mice compared with those of KCH^−/−^ mice, suggesting that *Hipk2*-KO reduces the number of activated fibroblasts but promotes collagen deposition. pERK^+^ cells were present in the majority of PanINs in both mouse lines, as expected from the requirement of oncogenic KRAS for PanIN transformation [35], but, compared to KC, in KCH^−/−^ we found a lower number of positive lesions with a lower staining intensity (Figs. 4E, F and 2E, F). Moreover, the number of lesions with pSTAT3^+^ cells and their staining intensity were lower in KCH^−/−^ mice than in KC mice (Fig. 4G, H), consistent with a reduced accumulation of stromal cells.

To confirm the different collagen deposition between KC and KCH^−/−^, we performed additional staining to detect both fibers and cells, *i.e*., Picrosirius red/Mayer’s Hemalum and Masson’s trichrome (Supplementary Fig. S4A). Comparable results were obtained with the two histological stains both supporting the different collagen deposition between KC and KCH^−/−^ mice. Furthermore, we detected increased mRNA levels of both collagen-1A1 and -1A2 in the total lysates of pancreata from KCH^−/−^ mice compared with those from KC mice (Supplementary Fig. S4B).

These findings indicate that in KCH^−/−^ mice, the pancreatic lesion phenotype is characterized by abundant collagen fibers and reduced number of αSMA^+^ and pSTAT3^+^ stromal cells while, in KC mice, a strong desmoplastic reaction is observed, as previously reported [13]. These divergent features resemble those recently observed in human pancreatic cancers, where different types of spatially confined sub-tumor microenvironments (sub-TMEs) have been described and associated with distinct tumor-suppressive or tumor-promoting functions. The first type of sub-TME, defined as "deserted", is characterized by a higher presence of collagen fibers and fewer cells, while the second, defined as “reactive”, is characterized by fewer fibers and a higher cell density [26]. Thus, we categorized the pancreatic lesions present in our KC and KCH^−/−^ mice based on deserted and reactive sub-TME phenotypes (Fig. 4I). This analysis showed that the prevailing sub-TME present in KC mice is the reactive, whereas that prevailing in KCH^−/−^ mice is the deserted one (Fig. 4L). These observations are consistent with the attenuated tumorigenicity observed in KCH^−/−^ mice and suggest that HIPK2 cooperates with oncogenic KRAS also in shaping the microenvironment of early pancreatic neoplasia.

### Pharmacological inhibition of HIPK2 prevents ADM and PanIN formation

Because our in vivo studies showed that genetic ablation of HIPK2 in KRAS^G12D^-expressing pancreas epithelial cells weakens oncogenic KRAS, desmoplastic reaction, and pancreatic tumorigenesis, we investigated the chemopreventive activity of the HIPK2 inhibitor 5-iodotubercidin (5-ITu) in the *Hipk2*-WT KC mice. Among the different HIPK2 inhibitors available, we choose the 5-ITu because it strongly inhibits HIPK2 kinase activity in vitro [36] and, most relevant for this study, it supports the replacement capacity of endocrine pancreatic beta-cells in rodents for diabetes treatment [23], indicating that 5-ITu is not detrimental, at least, for the endocrine pancreas.

**Fig. 5:**
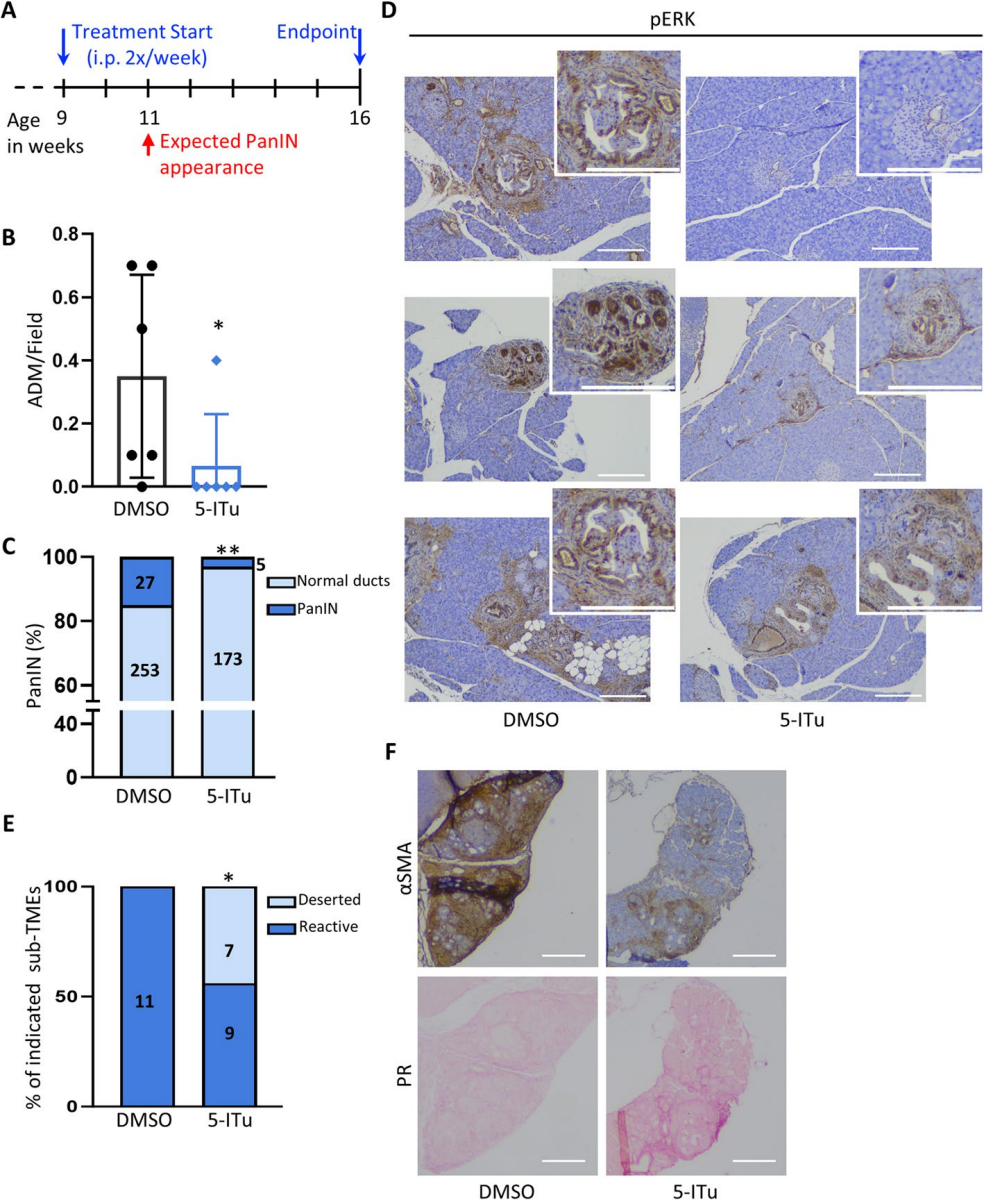
Inhibition of HIPK2 by 5-ITu prevents the onset of ADM and PanIN. **A** Schematic representation of the experimental design. **B** ADM in each visual field were counted in all DMSO and 5-ITu treated FVB-KC mice. The corresponding number of ADM per field for each mouse is reported in the scatter plot bar. Mean ± SD, Mann–Whitney’s test *P < 0.05. **C** Quantification of PanINs in the same DMSO and 5-ITu treated FVB-KC mice analyzed in B. The corresponding percentages are reported and the total number of normal ducts and PanINs counted is indicated inside the histograms. Fisher’s exact test, ** *P* < 0.01 **D** Representative images of pERK staining in DMSO and 5-ITu treated FVB-KC mice. For each image a 2x-magnified detail is shown in the upper-right corner. **E** The percentages of lesions with reactive and deserted sub-TMEs in DMSO and 5-ITu treated samples are reported. The total number of reactive and deserted subTMEs counted is indicated inside the histograms. Fisher’s Exact test, * *P* < 0.05. **F** Representative images of reactive and deserted sub-TMEs stained with αSMA (upper images) and Picrosirius Red (lower images). Scale bars are 200 µm

In the FVB strain, KC mice develop spontaneous PanINs earlier and faster than in the C57BL/6 strain, with pancreata beginning to show PanINs at 11 weeks of age [22]. Thus, to test the effect of HIPK2 inhibition by 5-ITu, we employed the FVB-KC mice starting the treatment at nine weeks of age and for the duration of two months, *i.e*., a time sufficient for the development of PanINs in over than 90% of control animals [22] (Fig. 5A). Twelve FVB-KC mice, six males and six females, born the same week from two littermates, were subdivided into two groups (three males and three females per group) and treated i.p. twice a week with 5-ITu or its solvent (DMSO) as control. During the treatment, no difference in body weight was observed between the two groups (Supplementary Fig. S5). After two months of treatment, all animals were euthanized, macroscopically analyzed, and their pancreas examined by H&E, PR, and IHC for pERK, αSMA, and pSTAT3, as described above. We observed macroscopic alterations, such as flushed pancreas and splenomegaly in five out of six control-treated mice; while only one out of six 5-ITu-treated mice showed splenomegaly (Supplementary Table S2). Histological evaluation of the 12 pancreata showed a strong reduction in ADM and PanIN development in the 5-ITu-treated mice, with five out of six pancreata being free from detectable alterations (Fig. 5B, C). Indeed, in the 5-ITu-group, only the mouse showing splenomegaly at the macroscopic evaluation presented ADM and PanINs while, in the control group, five out of six mice developed ADM and in four mice we also found PanINs. Next, we examined the features of PanINs by IHC and PR staining. In agreement with the observation made in KCH^−/−^ mice, pERK^+^ cells were detectable in the PanINs, but the intensity was much lower in the 5-ITu-treated mouse than in control mice (Fig. 5D). To assess the HIPK2 dependency of 5-ITu-mediated activity, organotypic cell cultures derived from KC and KCH^−/−^ C57BL/6 mice were treated with 5-ITu after KRAS pathway activation in response to EGFR stimulation. We observed that ERK phosphorylation is significantly reduced after 5-ITu treatment in KC organoids while no effect is detectable in KCH^−/−^ organoids (Supplementary Fig. S6), indicating that the 5-ITu activity on the Ras pathway is at least partially mediated by specific HIPK2 inhibition. Finally, the categorization into reactive and deserted sub-TMEs showed that all the lesions observed in the control mice belong to the reactive type; while half of the few lesions found in the 5-ITu-treated mouse were deserted (Fig. 5E, F), supporting a less aggressive phenotype. Taken together, these results indicate that pharmacological inhibition of HIPK2 in a mouse model of oncogenic KRAS-dependent preinvasive PDAC prevent ADM and its progression into aggressive PanINs.

## Discussion

PDAC is a highly lethal disease that arises from stepwise progression of preneoplastic lesions and preinvasive PanINs are often initiated by mutations in the *KRAS* gene. The increasing recognition of individuals who are at high-risk for developing PDAC has made research in chemoprevention an additional strategy to flank the development of new therapeutic approaches for invasive pancreatic cancers [37, 38]. In the current study, our analysis of genetic and pharmacological inactivation of HIPK2 in a mouse model of oncogenic KRAS-driven pancreatic cancer revealed that HIPK2 cooperates with the KRAS signaling for the development of ADM and PanIN lesions and the shape of desmoplastic reaction, and that HIPK2 inactivation can be explored as a preventive strategy. These findings are coherent with the observation that HIPK2 expression is maintained and even increased in human PDACs.

HIPK2 is involved in multiple signaling pathways, including those frequently altered in human cancers. Because of its apoptotic and growth-suppressing functions linked to TP53, its family members, and other pro-apoptotic factors in response to genotoxic damage, HIPK2 has been long considered a putative tumor-suppressor [14]. However, recent findings revealed a complex role of HIPK2 in different cancer types that strongly depends on tissue and cellular context and can determine either oncosuppressive or oncogenic effects [39]. Indeed, inactivating mutations of *HIPK2* are rare in human cancers, while conflicting results have emerged by IHC on different tumor types [19]. Another important aspect is related to the level of expression of HIPK2 that can determine the same effect in a opposite manner. Indeed, in a previous study on Panc1 and SW1990 PDAC cell lines, it has been shown that very high levels of HIPK2 expression, achieved through exogenous HIPK2 overexpression, inhibit, rather than supporting, ERK phosphorylation and induce tumor-suppressive effects by reducing cMyc and cMyc‐targeted glycolytic genes expression [40]. At this point, whether these opposite effects on ERK phosphorylation are due to upper and lower thresholds for HIPK2 protein levels or whether the effect observed in transfect cells mimic HIPK2 activation without reflecting cell physiology need to be directly evaluated. In apparent contrast with our model is the observation of intraindividual reduced levels of HIPK2 mRNA in tumor samples compared to relative para-tumor tissue [40]. However, when we analyzed the HIPK2 mRNA expression in our pancreatic cancer samples [41] (*i.e*., 26 out of 44 samples in which tumor mRNA was available) we did not find a consistent matching between the mRNA levels and the protein positivity by IHC (data not shown). In our opinion, overall, these results indicate a complex scenario in which post-translational mechanisms plays a major role in underlying the observed increase in HIPK2 positive cells. Nonetheless, we cannot exclude the possibility that the reported mRNA variability may contribute to develop specific tumor microenvironment that should be assessed by high resolution analyses such as spatial transcriptomic. In addition, tissue-specific effects and genetic background-related differences need to be clarified; however, some of the reported divergences are also most likely due to the low specificity of the Abs employed [27]. With this purpose, we recently reported that at molecular level HIPK2 physically participates to RAS/MAPK complex, cooperates with KRAS signaling, and associates with tumor progression in human colorectal cancers [19]. To validate this molecular crosstalk in a different tumor type driven by KRAS mutations and to verify whether the HIPK2/KRAS cooperation has a causal role in in vivo tumorigenicity, we chose PDAC, the prototype of oncogenic KRAS-driven cancers. First, we used a previously validated and highly specific anti-HIPK2 rat monoclonal Ab to perform WB analyses on PDAC-derived cell lines and IHC on biobanked PDAC samples. In contrast with previously reported data, generated with Abs that were not validated on human *HIPK2*-null cells [40, 42, 43], we found that *HIPK2* expression is maintained, and even increased in PDACs. This is consistent with *i*) cancer genome data retrieved from publicly available databases (*e.g*., COSMIC, cBioPortal) that report the presence of the wild-type HIPK2 gene in the majority of pancreatic cancers, *ii*) our previous data obtained on PDAC mRNA samples showing transcription of different *HIPK2* isoforms [41], and *iii*) the increased expression of *HIPK2* mediated by NRF2, a target of oncogenic RAS in different tumor cell types [44].

Based on these results, we generated a pancreas-specific *Hipk2*-KO in a well-characterized mouse model of KRAS^G12D^-dependent preinvasive PDAC [21]. Consistent with the data obtained in vitro in human colorectal cancer cells, we observed a significant attenuation of the oncogenic KRAS activity, as evaluated by both WB on whole pancreas lysates and IHC with anti-pERK Ab. Of relevance, this reduced oncogenic KRAS activity resulted in pancreatic phenotypes that mirror the observation made by direct mutant KRAS silencing through doxycycline inducible and reversible system [35] and, more recently, by mutant-KRAS-specific pharmacological inactivation [45]. In particular, pancreas-specific *Hipk2*-KO weakened both KRAS-dependent cell-autonomous and non-cell autonomous effects. Indeed, we observed both inhibition of ADM reversion and its subsequent progression into PanIN and activation of desmoplastic reaction, further supporting the contribution of HIPK2 in sustaining oncogenic KRAS signaling and in vivo tumorigenicity.

Interestingly, by evaluating the desmoplastic reaction on serially cut slices, we were able to identify the two spatially confined sub-TMEs (*i.e.*, reactive and deserted), recently described in human pancreatic cancers and differently associated with tumor-promoting and chemoprotective functions [26]. As expected, KC mice showed mainly the tumor-promoting, reactive sub-TME, while KCH^−/−^ mice were enriched in the deserted sub-TME. This is consistent with the less aggressive phenotype associated with this latter sub-TME in human PDAC and the reduced tumorigenicity linked to *Hipk2*-KO. Surprisingly, a further association between the prevalence of deserted sub-TME and the *Hipk2*-KO might be depicted, *i.e.*, the chemoprotective function. Indeed, neoadjuvant treated PDACs have been shown to exhibit a deserted-dominant TME compared to stage-matched treatment-naïve cases, indicating that upon chemotherapy, the less tumor-promoting deserted sub-TME also has chemoprotective effects. A similar consideration can be made on HIPK2, whose silencing in already transformed cancer cells has been consistently associated to resistance to chemotherapy [46], while its increased expression in stage II colorectal cancers has been shown to predict favorable response to adjuvant chemotherapy [47]. Thus, as reported by Grünwald and colleagues [26], also for HIPK2, response to chemotherapy and disease promotion appear to be independent functions. A deep characterization of these TME in premalignant phase of pancreatic cancer might be assessed to identify new potential preinvasive markers and specific protumorigenic functions.

Finally, we took advantage of the reduced oncogenic KRAS activities induced by *Hipk2*-KO and evaluated whether pharmacological inhibition of HIPK2 can be proposed as chemoprevention strategy for PanIN formation. In the last few years, several HIPK2 inhibitors have been developed [48–50]. However, none of them has been proven to be selective for this kinase because of the homology among the catalytic domains of the HIPK family members and the dual-specificity tyrosine-regulated kinase (DYRK) family members [51]. Among the small molecules that have been shown to inhibit the HIPK2 kinase activity [36] and that can be administered in vivo, we selected the 5-ITu because it has been shown to promote the replacement of islet β-cells from rodent, porcine, and human upon transplantation into immunocompromised mice for diabetes treatment [23]. When administered for two months to KC mice, we observed a complete prevention of both ADM and PanIN formation in five out of six of the 5-ITu treated mice while the opposite results (*i.e*., ADM and PanIN formation in five out of six animals) were obtained in the control, DMSO-treated mice, indicating a strong chemoprevention activity of 5-ITu in KRAS-driven pancreatic tumorigenesis. At this point, we can foresee two possible explanations, not necessarily mutually exclusive, for the stronger prevention activity of 5-ITu than *Hipk2*-KO. First, at variance from the genetic KO that was induced only in the epithelial, Pdx1-expressing cells, 5-ITu can inhibit HIPK2 activity in both epithelial and stromal cells, further impairing the non-cell autonomous activity of KRAS on desmoplastic reaction. This hypothesis is supported by previous findings showing that HIPK2 depletion can inhibit both mutant and wild-type RAS signaling activity [19, 20]. In addition, in the absence of HIPK2, two other members of its family, HIPK1 and HIPK3 can easily substitute for it [18]. A second possibility is linked to the broader kinase inhibition activity of 5-ITu than *Hipk2*-KO. KRAS pathway activation experiment conducted on KC and KCH^−/−^ murine pancreatic organoids indicated that the 5-ITu function is at least in part due to HIPK2 inhibition, nonetheless the 5-ITu is an ATP mimetic and a potent inhibitor of different kinases, including Adenosine kinase, Casein kinases 1 and 2, Protein kinases A and C, and Haspin [52, 53], opening the possibility that the 5-ITu-prevention function depends on inhibition of one or more of these kinases. At this point, we cannot rule out this possibility, which anyway would not be detrimental for further investigations of this small-molecule in PDAC chemoprevention; however, the reduced levels of pERK and the presence of deserted sub-TME in the mouse that develops ADM and PanIN despite 5-ITu treatment support the conclusion that 5-ITu is acting, at least in part, on the HIPK2/KRAS axis.

In summary, this study is the first to analyze the role of molecular cooperation of HIPK2 with KRAS signaling, already associated to colorectal cancer, in the KRAS-driven tumorigenesis of the pancreas using mice with tissue-specific expression of oncogenic KRAS and *Hipk2*-KO. We provide evidence that HIPK2 contributes to sustaining effective oncogenic KRAS signaling for both cell-autonomous (*i.e*., blockade of ADM reversion and PanIN development) and non-cell autonomous actions (*i.e*., shape of desmoplastic reaction). The current study opens the way for new chemoprevention approaches for cohorts of individuals who are at high-risk for developing PDAC.

## Supplementary Information

Supplementary Material 1.

## Data Availability

Data that support the findings of this study are available from the corresponding author upon reasonable request.

## Abbreviations

HIPK2: Homeodomain-Interacting Protein Kinase 2
PDAC: Pancreatic ductal adenocarcinoma
TME: Tumor microenvironment
KC: Pdx1-Cre;LSL-KRas^G12D/+^
KCH^−^^/^^−^: Pdx1-Cre;Hipk2^flox/flox^;LSL-KRas^G12D/+^
5-ITu: 5-IodoTubercidin
ADM: Acinar-to-ductal metaplasia
PanIN: Pancreatic intraepithelial neoplasms
ERK1/2: Extracellular signal-regulated kinases 1 and 2
MAPK: Mitogen activated protein kinase
KO: Knockout
WT: Wild type
αSMA: α-Smooth muscle actin
STAT3: Signal transducer and activator of transcription 3
WB: Western Blot
FFPE: Formalin-fixed paraffin-embedded
H&E: Hematoxylin and eosin
Ab: Antibody
i.p.: Intraperitoneally
BW: Body weight
DMSO: Dimethyl sulfoxyde
PCR: Polymerase Chain Reaction
RT-qPCR: Quantitative reverse transcription PCR
PR: Picrosirius Red
IHC: Immunohistochemistry
min: Minute
IRE: Regina Elena Cancer Institute
Gapdh: Glyceraldehyde-3-phosphate dehydrogenase
mo: Month
