# Supplementary material for: Inactivation of HIPK2 attenuates KRASG12D activity and prevents pancreatic tumorigenesis
Source: J Exp Clin Cancer Res. 2024 Sep 28;43:265. doi: 10.1186/s13046-024-03189-3 (PMC11437985; doi:10.1186/s13046-024-03189-3)
Supplement: Supplementary file 1 — Supplementary Material 1. [file 13046_2024_3189_MOESM1_ESM.pdf]

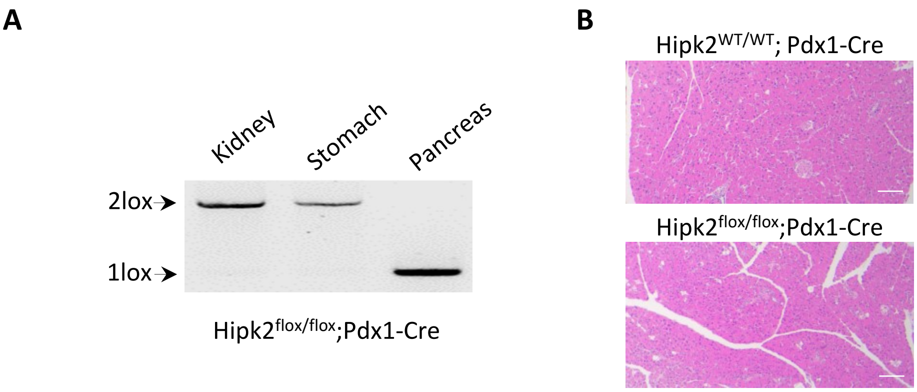

Supplementary Figure S1.

A, Genotypization for *Hipk2* recombinant allele in Hipk2$^{flox/flox}$;Pdx1-Cre in the indicated organs. The upper bands are the PCR products of the floxed gene (2lox), the lower band is the PCR product of the recombined allele (1lox). **B**, Representative images of H&E stained pancreatic tissues from Hipk2$^{flox/flox}$;Pdx1-Cre and Hipk2$^{wt/wt}$;Pdx1-Cre mice. Scale bars are 200μm.

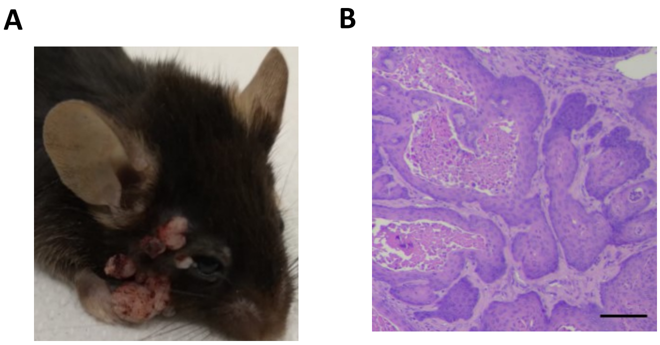

Supplementary Figure S2.

A, Photo of papilloma on the muzzle of a C57BL6 KC mouse. **B**, Representative IHC of the papilloma. Scale bar is 200μm.

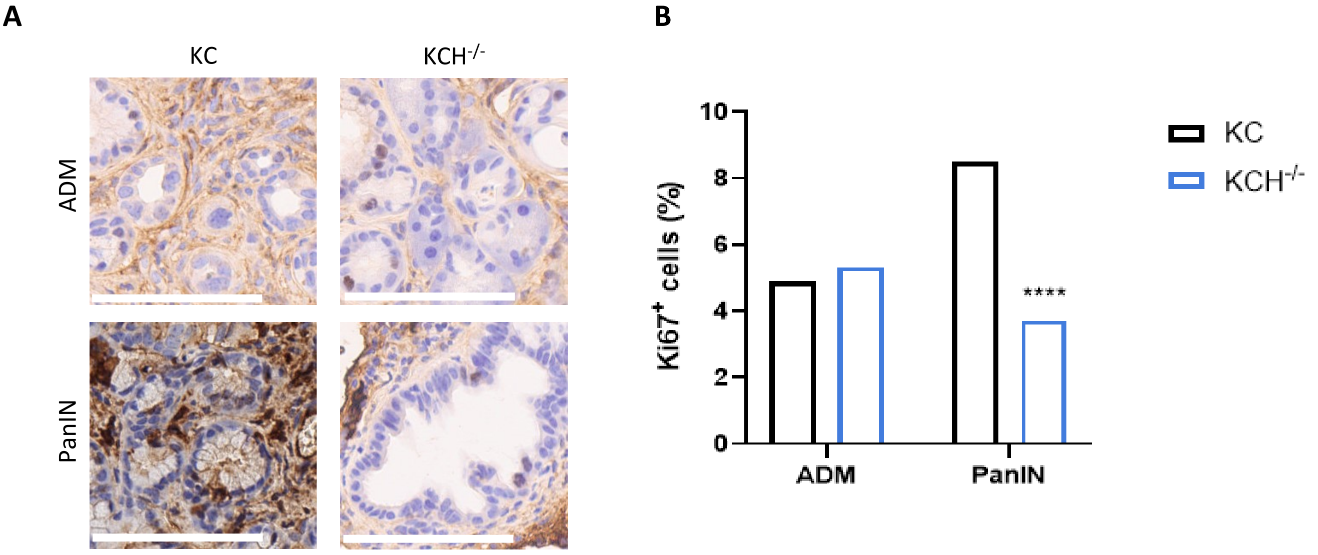

Supplementary Figure S3.

A, Representative images of Ki67 immunostaining in ADM and PanIN from KC and KCH$^{-/-}$ mice. Scale bars are 100μm. **B,** Ki67^{+} cells in ADM and PanIN ducts were counted in KC and KCH$^{-/-}$ pancreata on a total of at least 800 cells for each condition. The resulting percentages are reported as histograms. Fisher's exact test, **** P<0.0001.

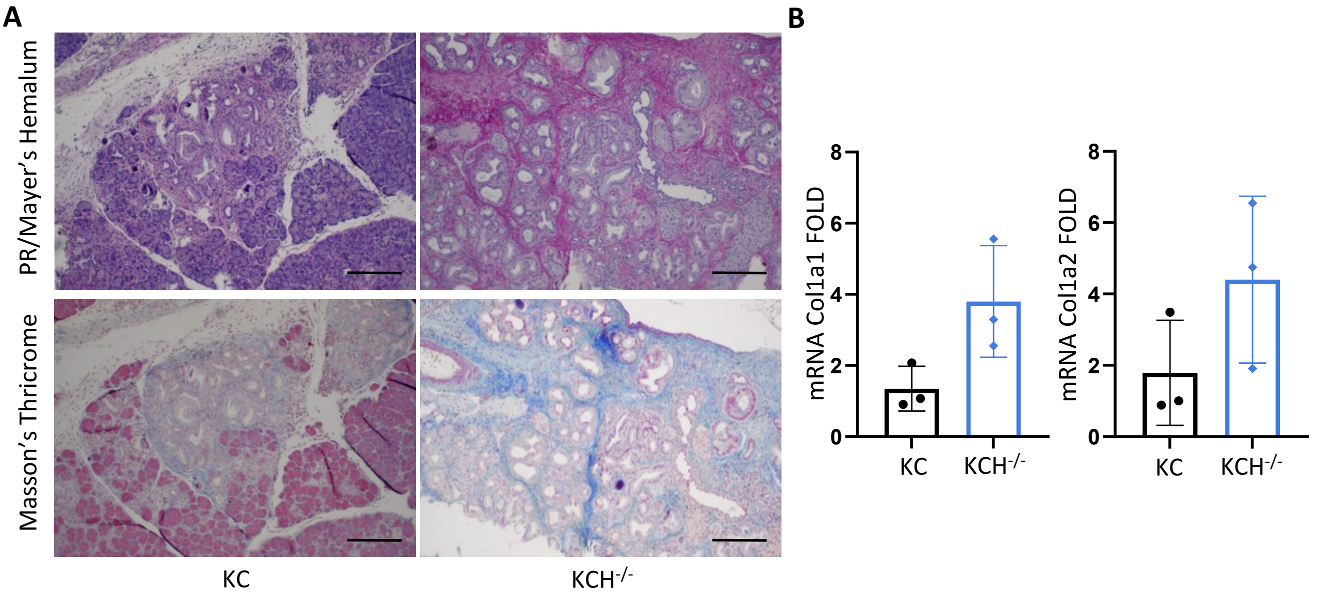

Supplementary Figure S4.

A, Representative images of pancreatic tissues from KC and KCH$^{-/-}$ mice stained with Picrosirius Red (PR)/Mayer's Hemalum (upper panels) and Masson's Thricrome (lower panels). Scale bars are 200μm. **B**, Collagen I mRNA expression analysis. Col1a1 (left graph) and Col1a2 (right graph) mRNA expression is shown as fold difference (2^-ΔΔCt) in scatter plot bars.

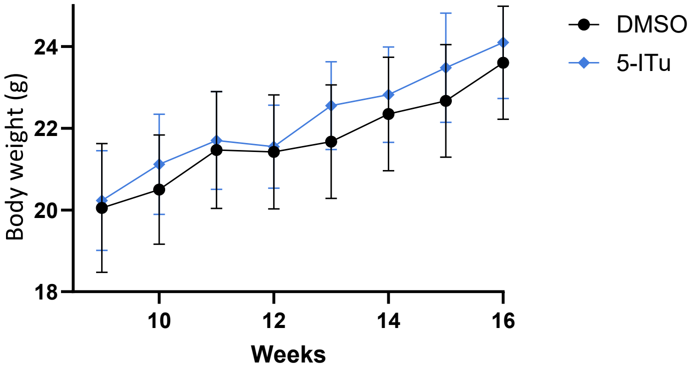

Supplementary Figure S5.

Growth curve of FVB KC mice treated with 5-ITu or DMSO. Mean ± SD

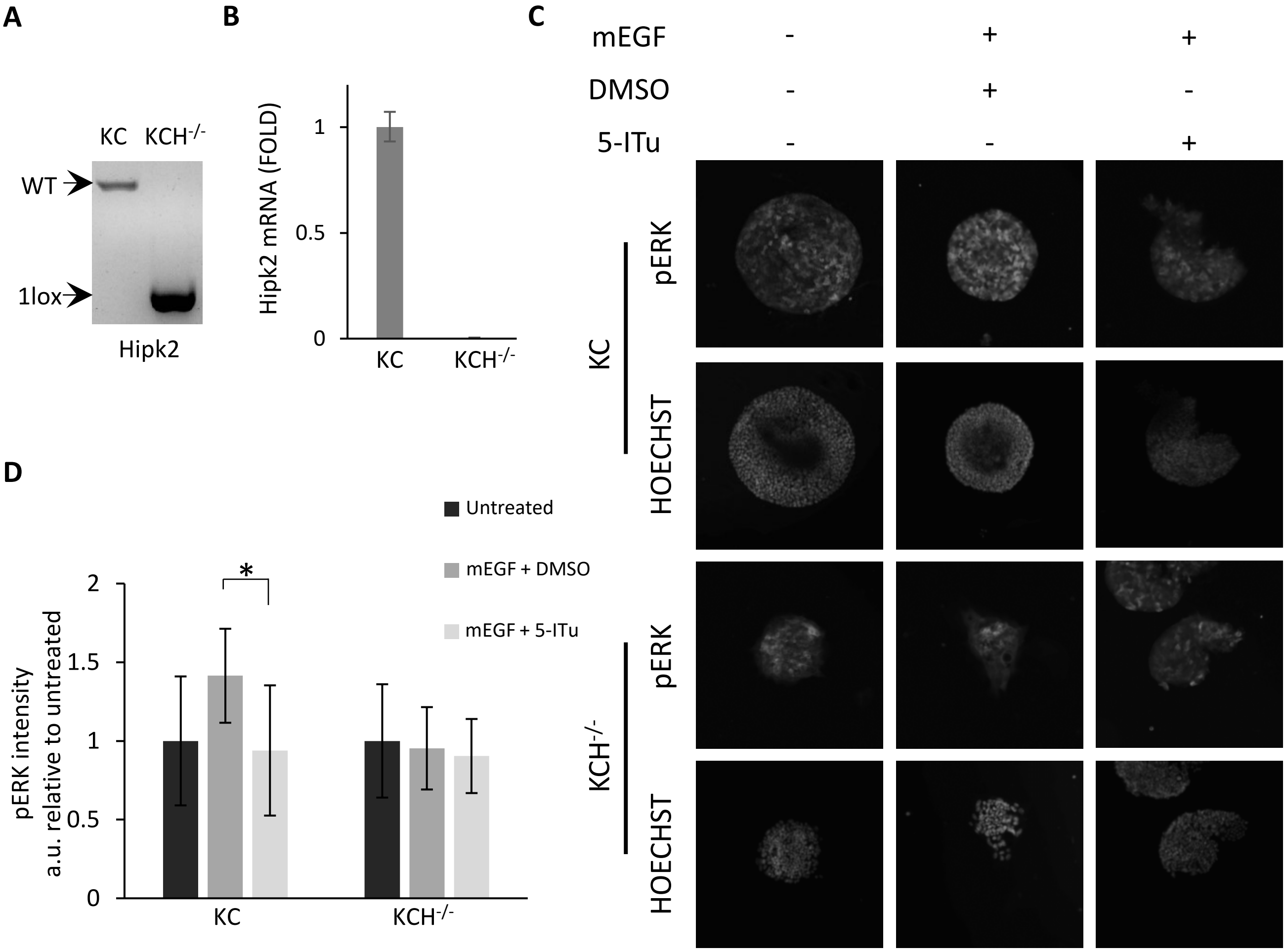

Supplementary Figure S6.

5-ITu effect on KRAS pathway activation in KC and KCH$^{-/-}$ pancreatic murine organoids.

A, Validation of Hipk2^{flox} allele recombination in KCH$^{-/-}$ organoids. Genotypization for *Hipk2* recombinant allele in KCH$^{-/-}$ was assessed by PCR analysis. KC sample was used as control. The upper bands are the PCR products of the floxed (2lox) or WT genes, the lower band is the PCR product of the recombined allele (1lox). **B,** RT-qPCR analysis on HIPK2 mRNA extracted from KC and KCH$^{-/-}$ organoids. 2^-ΔΔCt (Fold) relative to KC are shown in the histogram. **C,** Representative images of pERK intensity in KC and KCH$^{-/-}$ organoids cultured with the indicated treatments and stained for anti-pERK and HOECHST. Images were acquired using Nikon Eclipse Ti2 inverted fluorescence microscope. **D**, pERK intensity was assessed as arbitrary unit (a.u.) for each organoid using ImageJ software. Histograms show the intensity level of treated organoids relative to untreated control as mean ± SD. Student's t test *p<0.01.

Antibodies	Manufacturer	Cat. Number	RRID	IHC	WB
αSMA	Abcam	Ab124964	AB_11129103	Ag R. 1:1000 - 2h	-
ERK	Cell Signaling	#4695	AB_390779	-	1:1000 - O.N.
HIPK2	Provided by L.M. Schmitz [27]	5C6	-	1:50 - 30min	1:200 - O.N.
HSP70	Sigma-Aldrich	SAB4200714	-	-	1:1000 - O.N.
pERK	Cell Signaling	#4370	AB_2315112	Ag R. 1:100 - O.N.	1:1000 - O.N.
pSTAT3	Cell Signaling	#9145	AB_2491009	Ag R. 1:100 - O.N.	-
Ki67	Cell Signaling	#12202	AB_2620142	Ag R. 1:200 – 1h	-
CD3	Abcam	Ab16669	AB_443425	Ag R. 1:100 - O.N.	-
Cleaved Caspase 3	Cell Signaling	#9661	AB_2341188	Ag R. 1:200 - O.N.	-
IgG mouse-HRP	Cell Signaling	#7076	AB_330924	-	1h RT
IgG rabbit-HRP	Cell Signaling	#7074	AB_2099233	-	1h RT
IgG rat-HRP	Thermofisher	#31470	AB_228356	-	1h RT
IgG rabbit-488	Alexa Fluor™	A-21206	AB_2535792	1h 37°C (IF)	-

O.N.: overnight; Ag R.: Antigen Retrieval; RT: Room Temperature

Method	Gene	Forward Primer	Reverse Primer
PCR	HIPK2	GACTTAGTGCCCTCTTGCCC	CTGTTTCTCCATGGACAGTGGGTT
PCR	Pdx1-CRE	ATGCTTCTGTCCGTTTGCCG	TGAGTGAACGAACCTGGTCG
PCR	KRAS-LSL-G12D	AGCTAGCCACCATGGCTTGAGTAAGTCTGCA	CTCTTGCCTACGCCACCAGCTC
PCR	KRAS WT/G12D	GTCTTTCCCCAGCACAGTGC	CTCTTGCCTACGCCACCAGCTC
RT-qPCR	HIPK2	CACAAACACAGGGTTTACCAG	TGGTGTCTTTAGCCTCCAC
RT-qPCR	ACTA2	GTCCCAGACATCAGGGAGTAA	TCGGATACTTCAGCGTCAGGA
RT-qPCR	Col1A1	GCTCCTCTTAGGGGCCACT	CCACGTCTCACCATTGGGG
RT-qPCR	Col1A2	GTAACTTCGTGCCTAGCAACA	CCTTTGTCAGAATACTGAGCAGC
RT-qPCR	GAPDH	AGGTCGGTGTGAACGGATTTG	TGTAGACCATGTAGTTGAGGTCA

Supplementary Table S1
List of Antibodies and PCR oligos.

MouseID	Sex	Treatment	Pancreas Histology	Other pathological features
08029	♀	5% DMSO	ADM, PanIN	Splenomegaly; Spotted Lungs
08039	♀	5% DMSO	ADM, PanIN	Lung Cancer; Flushed Pancreas
08026	♂	5% DMSO	ADM, PanIN	Spotted Lungs
08027	♂	5% DMSO	Normal pancreatic tissue	Spotted Lungs
08008	♂	5% DMSO	ADM	nothing to report
08041	♀	5% DMSO	ADM, PanIN	nothing to report
08030	♂	5-ITU	Normal pancreatic tissue	nothing to report
08031	♂	5-ITU	Normal pancreatic tissue	nothing to report
08032	♂	5-ITU	Normal pancreatic tissue	nothing to report
08022	♀	5-ITU	Normal pancreatic tissue	nothing to report
08014	♀	5-ITU	Normal pancreatic tissue	nothing to report
08012	♀	5-ITU	ADM, PanIN	Splenomegaly

Supplementary Table S2. Clinical spectrum of disease in DMSO or 5-ITu treated KC mice.
